# Quantitative analysis of MRI‐guided radiotherapy treatment process time for tumor real‐time gating efficiency

**DOI:** 10.1002/acm2.13030

**Published:** 2020-10-22

**Authors:** Lorenzo Placidi, Davide Cusumano, Luca Boldrini, Claudio Votta, Veronica Pollutri, Marco Valerio Antonelli, Giuditta Chiloiro, Angela Romano, Viola De Luca, Francesco Catucci, Luca Indovina, Vincenzo Valentini

**Affiliations:** ^1^ Dipartimento di Diagnostica per Immagini, Radioterapia Oncologica ed Ematologia UOC Radioterapia Oncologica Fondazione Policlinico Universitario “A. Gemelli” IRCCS Roma Italy; ^2^ Università Cattolica del Sacro Cuore Rome Italy

**Keywords:** gating efficiency, MR‐guided radiotherapy, time analysis

## Abstract

**Purpose:**

Magnetic Resonance‐guided radiotherapy (MRgRT) systems allow continuous monitoring of therapy volumes during treatment delivery and personalized respiratory gating approaches. Treatment length may therefore be significantly affected by patient’s compliance and breathing control. We quantitatively analyzed treatment process time efficiency (*T_E_*) using data obtained from real‐world patient treatment logs to optimize MRgRT delivery settings.

**Methods:**

Data corresponding to the first 100 patients treated with a low T hybrid MRI‐Linac system, both in free breathing (FB) and in breath hold inspiration (BHI) were collected. *T_E_* has been computed as the percentage difference of the actual single fraction’s total treatment time and the predicted treatment process time, as computed by the TPS during plan optimization.

Differences between the scheduled and actual treatment room occupancy time were also evaluated. Finally, possible correlations with planning, delivery and clinical parameters with *T_E_* were also investigated.

**Results:**

Nine hundred and nineteen treatment fractions were evaluated. *T_E_* difference between BHI and FB patients’ groups was statistically significant and the mean *T_E_* were 42.4%, and −0.5% respectively.

No correlation was found with *T_E_* for BHI and FB groups. Planning, delivering and clinical parameters classified BHI and FB groups, but no correlation with *T_E_* was found.

**Conclusion:**

The use of BHI gating technique can increase the treatment process time significantly. BHI technique could be not always an adequate delivery technique to optimize the treatment process time. Further gating techniques should be considered to improve the use of MRgRT.

## 1 INTRODUCTION

Cancer is one of the leading causes of death throughout the world. Each year, 4.6 million new cancer cases are diagnosed in the WHO European Region and 2.1 million people die from cancer.^1^

Radiotherapy (RT) treatment is always playing always a greater role and RT cancer treatments have been developed from relatively simple processes into very complex procedures, recently introducing several new technologies and delivery techniques into clinical practice.^2^

One of the most innovative technologies is represented by Magnetic Resonance‐guided Radiotherapy (MRgRT) hybrid units, that combine high dose distribution conformality and online adaptation with high quality positioning imaging.^3^, ^4^

The MRIdian Linac system (ViewRay Inc., Mountain View, California, US) has been the first example of a hybrid RT machine authorized for clinical treatments, joining a 0.35 Tesla MRI on board scanner with a 6 MV flattening filter free (FFF) Linac system.^3^ One of the most significant advantages of this technology is represented by the possibility to monitor online the target during the whole radiotherapy treatment, using sagittal MR images acquired in cine modality with 4 frames/s. This leads to evident advantages especially in the case of the irradiation of movable targets, successfully integrating the motion management strategies to date available in current radiotherapy such as respiratory gating with surface markers or surface motions control.^5^ However, these methods are exposed to intra‐fractional changes in the relationship between the internal tumor/OARs motion and the external surface related signal.^6^, ^7^ To reduce the uncertainties of the issue of gating with a surrogate signal, internal markers could be implanted inside or close to the target lesion: nevertheless, this process is invasive for the patient and time consuming.

**Fig. 1:**
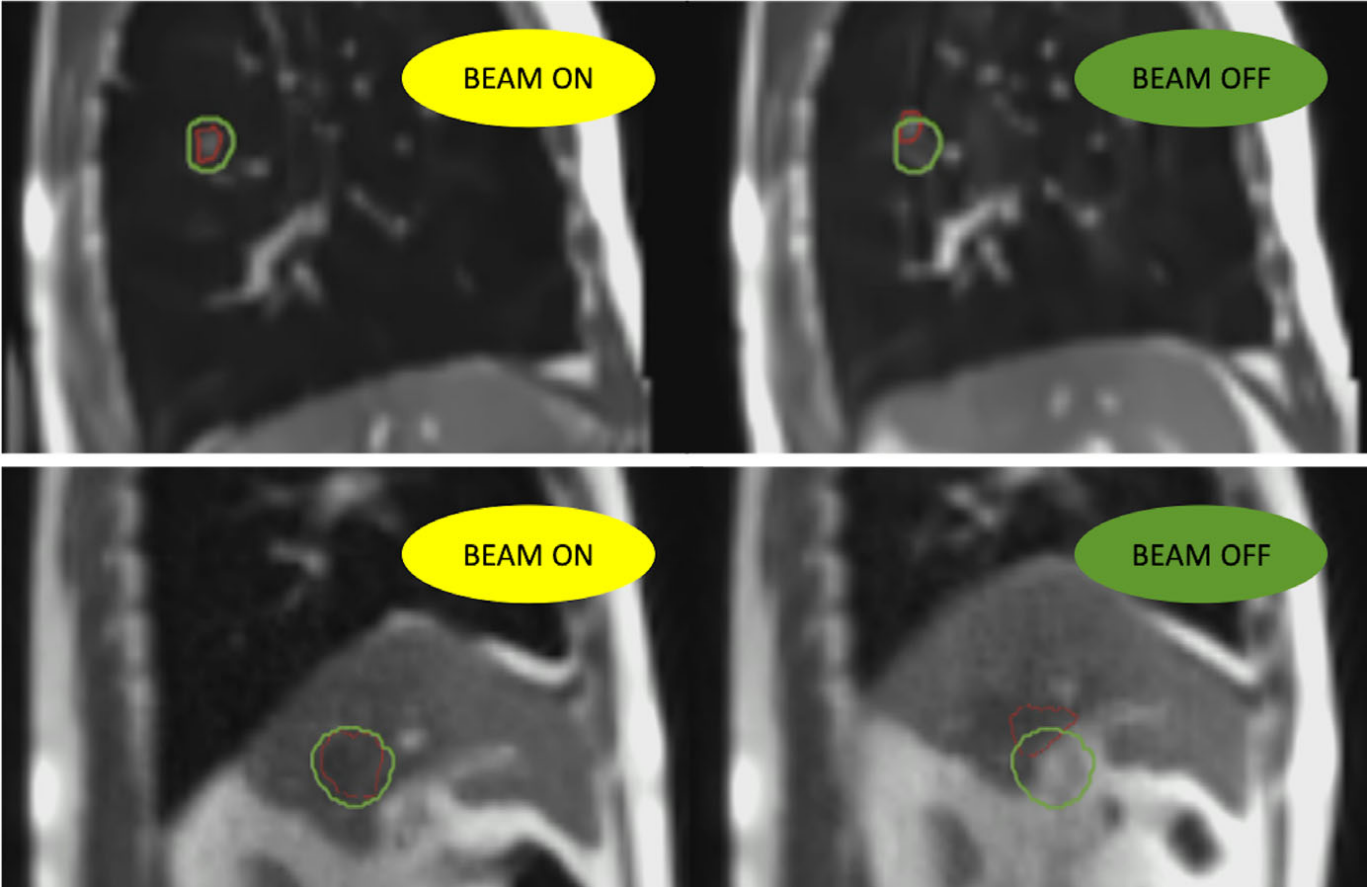
Real‐time sagittal MRI acquisition during treatment delivery (top images is a lung case, bottom images is a liver case): target (red) is irradiated (BEAM ON) only when it is located within the boundary (green). If a defined percentage of the target is outside of the boundary, delivery is automatically interrupted (BEAM OFF).

Thanks to the use of a real‐time sagittal MRI acquisition with a temporal frequency of 4 frames per second, the target is irradiated, as shown in Fig. 1, only when it is located within a predefined boundary, usually defined as a geometric expansion of the target structure itself.^8^

During treatment simulation, the breathing modality is also evaluated, and the most appropriate delivery technique is chosen between breath hold (BH) or free breathing (FB): this decision is generally treatment site dependent. Treatment sites where breathing motion leads to a relevant variation of the lesion position, such as lung, liver, pancreas, adrenal gland and (some) lymph nodes are usually treated in BH. Nevertheless, for example, soma central lung lesions can be treated in free breathing since the lesion excursion due to the breathing path is negligible. On the other hand, treatment sites where breathing motion does not affect the lesion position, such as rectum, cervix, prostate and lymph nodes, are usually treated in FB. A secondary parameter that is taken into account to select the most appropriate delivery technique, but not less important, is the patient’s compliance. In particular, for the BH delivery technique, is extremely important to accurately examine and evaluate the simulation MRI images dataset: if the patient is not suitable to proceed a BH treatment, another delivery technique (FB) is considered, as well as to move the patient to a standard linear accelerator. Despite the advantages offered by real‐time motion monitoring, there still are some concerns on the fact that the beam delivery time can be greatly prolonged in the case of inspiratory BH gating treatment.

The efficiency of RT delivery on mobile targets is extremely important in this context, as the average beam delivery time for respiratory‐gated irradiation can be two to five times longer than the free breathing one for equivalent fractionation.

As treatment room throughput and efficiency management is also crucial for this innovative technology, some experiences have been published to evaluate treatment process time,^9^, ^10^ considering the system log data.^11^

Recording these parameters has a huge impact also for optimizing the daily treatment room schedule into specific time slots.^9^, ^10^

To the best of our knowledge, treatment process time efficiency for the MRgRT system has not yet been analyzed.

This study aims to evaluate treatment process time efficiency based on our clinical experience, and to propose a new treatment room management approach in the case of BHI or FB conditions.

## 2 MATERIALS AND METHODS

### 2.A Patients database

A sample of consecutive patients treated in our institution with the MRIdian MR‐Linac (ViewRay Inc, Mountain View, CA, USA) was considered for this analysis. Patients have been grouped based on of the disease site: lung, liver, pancreas, adrenal gland, lymph node, rectum, cervix and prostate. Both BHI and FB patients were considered for this analysis.

### 2.B Treatment planning

All patients included in the study underwent a simulation MRI (0.35 T) and CT scan (GE, Optima CT580 W, HiSpeed DX/I Spiral), acquired sequentially on the same day. Simulation MRI scan is performed with a TRUFI sequence with a steady state precession sequence, image resolution of 1.5 × 1.5 × 1.5 mm^3^ and acquisition time of 25 or 175 s. Simulation CT scan, with a slice thickness of 2.5 mm, is acquired in the same position and with the same immobilization and positioning system used in the simulation MRI. During the MRI simulation, one or more sagittal cine MRI (4 frames/s) is also acquired to further define if the treatment will be performed in BH of FB delivery technique, considering both clinical/dosimetric requirements and patient’s compliance. Intensity modulated radiation therapy (IMRT) step and shoot treatment plans were calculated using the MRIdian treatment planning system (TPS).

For each treatment plan, the dose was prescribed according to the planning target volume (PTV) and normalized to the 50% of the PTV (in case of homogeneous dose prescription) or to the 80% isodose line (in case of inhomogeneous dose prescription).

MRI‐Linac can be used either for standard treatment delivery or in adaptive online modality. Adaptive online modality consists in adapting the treatment plan every day on the basis of the inter‐fraction changes in internal anatomy, ensuring the best dose distribution.^12^ For each patient treated with online adaptive modality, the evaluation of the daily anatomy could have led to a newly optimized plan, which is re‐optimized just some minutes before fraction delivery. For the patients not candidate for online adaptive delivery, offline replanning was performed, if necessary.

### 2.C Real‐time gating magnetic resonance‐guided radiotherapy system

Two key parameters have been defined for the sagittal cine MRI (4 frames/s) image acquisition prior to treatment delivery start:

1. Gating boundary, defined as a margin from CTV which will take into account target intra‐fraction maximum allowed motion. It depends on anatomical site and patients’ characteristics but is generally set to 3–5 mm.
2. Maximum percentage of gating target volume (ROI%), defined as the maximum allowed percentage of the target volume that should be outside the defined boundary to stop beam delivery. When this threshold value is exceeded, the beam is automatically interrupted. Also ROI% appears to be strongly dependent on the anatomical site and patients’ characteristics but it is generally set based on the target structure volumes (V < 8cc, 5 cc < V<20cc, V> 20 cc), respectively to 3–5–8%.

Both the described parameters are applied for BH and FB patients’ image acquisition and treatment. This is one of the most important novelties of the MRgRT system that enables the real‐time tumor tracking and beam gating also to FB patients, increasing the accuracy of the tumor intra‐fraction tracking by preventing any possible bulk motion of the patient on the treatment couch.

### 2.D Treatment process time efficiency

Beam on delivery time has already been defined by Suzuki et al.,^10^ for passive scattering or spot scanning proton therapy.

Yoshimura et al.,^11^ redefined the beam on delivery time taking into account not only the number of fields per session and the CTV volume, as proposed by Suzuki et al.,^10^ but also including the possible use of the gating approaches.

Total treatment time and mechanical IMRT step and shoot treatment time (i.e. Multi Leaf Collimator (MLC) and Gantry rotation) were also considered for this study. Machine and TPS log data have been used in order to extract the aforementioned variables.

In particular, beam on delivery time is defined as:

$$T_{\mathrm{Bod}}\left(X,V,R\right)$$

where *X* is the number of fields per session, *V* is the CTV in cc and *R* is the gating function (*R* = 1 with active gating and *R* = 0 without).

*T_Bod_* (*X*,*V*,*R*) is the actual time of beam delivery, nominally counted when MU are delivered.

The mechanical treatment time ($T_{G})$ is defined for gantry rotation and MLC configuration respectively as:

$$T_{G}\left(X,V\right)\;\text{and}\;T_{\mathrm{MLC}}\left(X,V\right).$$

In the delivery treatment log data, actual beam on time *T_Abo_* (*X*,*V*,*R*) is recorded as:

$$T_{\mathrm{Abo}}\left(X,V,R\right)=T_{\mathrm{Bod}}\left(X,V,R\right)+T_{\mathrm{MLC}}\left(X,V\right)+gating.$$

The actual total treatment process time ($T_{\mathrm{Attp}})$, is defined as:

$$T_{\mathrm{Attp}}\left(X,V,R\right)=T_{\mathrm{Abo}}\left(X,V,R\right)+T_{G}\left(X,V\right).$$

Finally, treatment process time efficiency (defined as treatment efficiency *T_E_* (*X*,*V*,*R*)) is expressed as:

$$T_{E}\left(X,V,R\right)=\frac{T_{\mathrm{Attp}}\left(X,V,R\right)‐T_{\mathrm{TPSttp}}\left(X,V,R\right)}{T_{\mathrm{TPSttp}}\left(X,V,R\right)}$$

where *T_Attp_* (*X*,*V*,*R*) is the actual total treatment process time (for each single fraction) and *T_TPSttp_* (*X*,*V*,*R*) is the predicted treatment process time computed by the TPS during the plan optimization. Negative values of *T_E_* (*X*,*V*,*R*) mean that the TPS has overestimated the treatment process time needed. *T_E_* (*X*,*V*,*R*), defined as percentage, has been analyzed for each single fraction of all the patients enrolled in the study and displayed in a Whisker plot.^13^

Welch two sample t‐test has been performed to evaluate the difference between the considered patients’ groups (BHI vs FB).

### 2.E Treatment slot time

In our current daily treatment room schedule, adaptive treatments have a 1‐h time slot, stereotactic Body Radiation Therapy (SBRT) treatments have a 45‐min time slot and the remaining treatments (long course, conventional fractionation) have a 30‐min time slot. The time needed for the pretreatment process (PTP) should be taken into account to quantitatively analyze treatment time, evaluating treatment slot time suitability and management.

Pretreatment process presents different phases:

1. Patient ready for positioning: the time required to let the patient inside treatment vault and check for the absence of ferromagnetic objects;
2. Patient positioning: immobilization and positioning systems, MRI coils, mirror adjustment to support the patient during gating treatment (if foreseen);
3. Positioning correction: acquisition of daily positioning MRI and couch shift application;
4. Adaptive process: target and OARs re‐contouring, plan re‐optimization and evaluation, QA (strongly dependent on patient daily anatomy and operators).

Total PTP time, for the different treatment types, has been evaluated among the different treatments included in the study.

### 2.F Clinical, planning and delivering parameters

In order to analyze possible correlation with treatment process time efficiency, several clinical, planning and delivering parameters were included in the study: clinical target volume (CTV) volume (cc), age, sex, number of fractions, dose per fraction, treatment modality (Standard fractionation/hypofractionation and Adaptive/no‐Adaptive), number of beams and segments have been considered as planning and clinical parameters.

Also, parameters relative to delivery were included: treatment boundary value for gating purposes, percentage of target volume outside the boundary up to which the delivery was allowed and the number of completion fractions.

To summarize, this study has three principal aims:

- treatment process time efficiency — comparison of patients treated in BHI and FB conditions, introducing an efficiency score (*T_E_*) that essentially indicates how close the treatment time provided by the TPS (total, mechanical and beam on) is to the actual delivery one;
- treatment slot time suitability — quantitative assessment of the duration of treatment slots foreseen for BHI patients, in order to optimize the daily treatment room scheduling and the total number of procedures;
- clinical, planning and delivery parameters correlation — investigating possible correlations between treatment process efficiency and clinical/planning/delivery parameters

## 3 RESULTS

The first 100 patients treated in our institution between June and November 2019, corresponding to 919 treatment fractions, were considered for this analysis.

Table 1 contains the distribution of patients for each category with (BHI) and without active respiratory motion management (FB).

**Table 1:** Patients’ characteristics, divided for treatment site, sex and age.

	N	[%]	Age	
			Mean	Range
Sex				
Male	48	48	70	40–96
Female	52	52	61	33–94
Categories				
BHI				
Lung	12	23%	66	50–81
Liver	23	43%	64	43–80
Pancreas	8	15%	70	59–81
Adrenal gland	3	6%	63	58–72
Lymph node	6	11%	63	47–78
Cervix	1	2%	43	―
FB				
Rectum	13	28%	65	40–85
Cervix	12	25%	57	33–94
Prostate	13	28%	74	66–85
Lymph nodes	8	17%	67	49–85
Lung	1	2%	78	—

### 3.A Treatment process time efficiency

Results (mean and standard deviation, SD) of the actual beam on time (*T_Abo_* (*X*,*V*,*R*)), actual total treatment process time (*T_Attp_* (*X*,*V*,*R*)), predicted treatment process time as computed by the TPS (*T_TPSttp_* (*X*,*V*,*R*)) and treatment process time efficiency (*T_E_* (*X*,*V*,*R*)) for patients treated with (BHI) and without (FB) active respiratory control, are reported in Table 2.

**Table 2:** actual beam on time (*T_Abo_* (*X*,*V*,*R*)), actual total treatment process time (*T_Attp_* (*X*,*V*,*R*)), predicted treatment process time computed by the TPS (*T_TPSttp_* (*X*,*V*,*R*)) and treatment efficiency (*T_E_* (*X*,*V*,*R*)) results for patients treated with and without active breathing control approaches.

Categories	*T_Abo_* (*X*,*V*,*R*) (min)	*T_Attp_* (*X*,*V*,*R*) (min)	*T_TPSttp_* (*X*,*V*,*R*) (min)	*T_E_* (*X*,*V*,*R*) (%)
	(Mean ± SD)	(Mean ± SD)	(Mean ± SD)	(Mean ± SD)
BHI				
Lung	14.6 ± 3.8	17.3 ± 3.9	10.9 ± 1.6	55.1 ± 38.6
Liver	11.9 ± 4.7	14.4 ± 5.1	10.2 ± 2.4	42.5 ± 36.1
Pancreas	12.6 ± 6,.1	15.5 ± 6.4	11.2 ± 2.7	35.1 ± 50.2
Adrenal gland	14.6 ± 2.4	18.2 ± 2.4	14.9 ± 2.5	43.4 ± 12.3
Lymph node	11.3 ± 4.0	13.7 ± 4.0	10.8 ± 1.9	32.6 ± 36.6
Cervix	6.8 ± 1.9	9.4 ± 2.1	4.1	40.2 ± 31.7
ALL	12.6 ± 4.9	15.3 ± 5.2	10.5 ± 2.5	42.4 ± 40.8
FB				
Rectum	9.2 ± 1.9	11.8 ± 2.1	11.9 ± 2.5	−2.3 ± 4.2
Cervix	10.8 ± 3.1	13.5 ± 3.2	13.7 ± 3.9	−2.0 ± 5.7
Prostate	9.5 ± 3.4	12.2 ± 3.5	11.9 ± 3.6	2.8 ± 9.0
Lymph nodes	10.1 ± 3.9	12.6 ± 4.3	12.3 ± 3.8	7.0 ± 9.3
Lung	7.6	9.7	9.64	1.4
ALL	10.4 ± 3.2	13.1 ± 3.4	11.6 ± 3.7	−0.5 ± 6.6

**Fig. 2:**
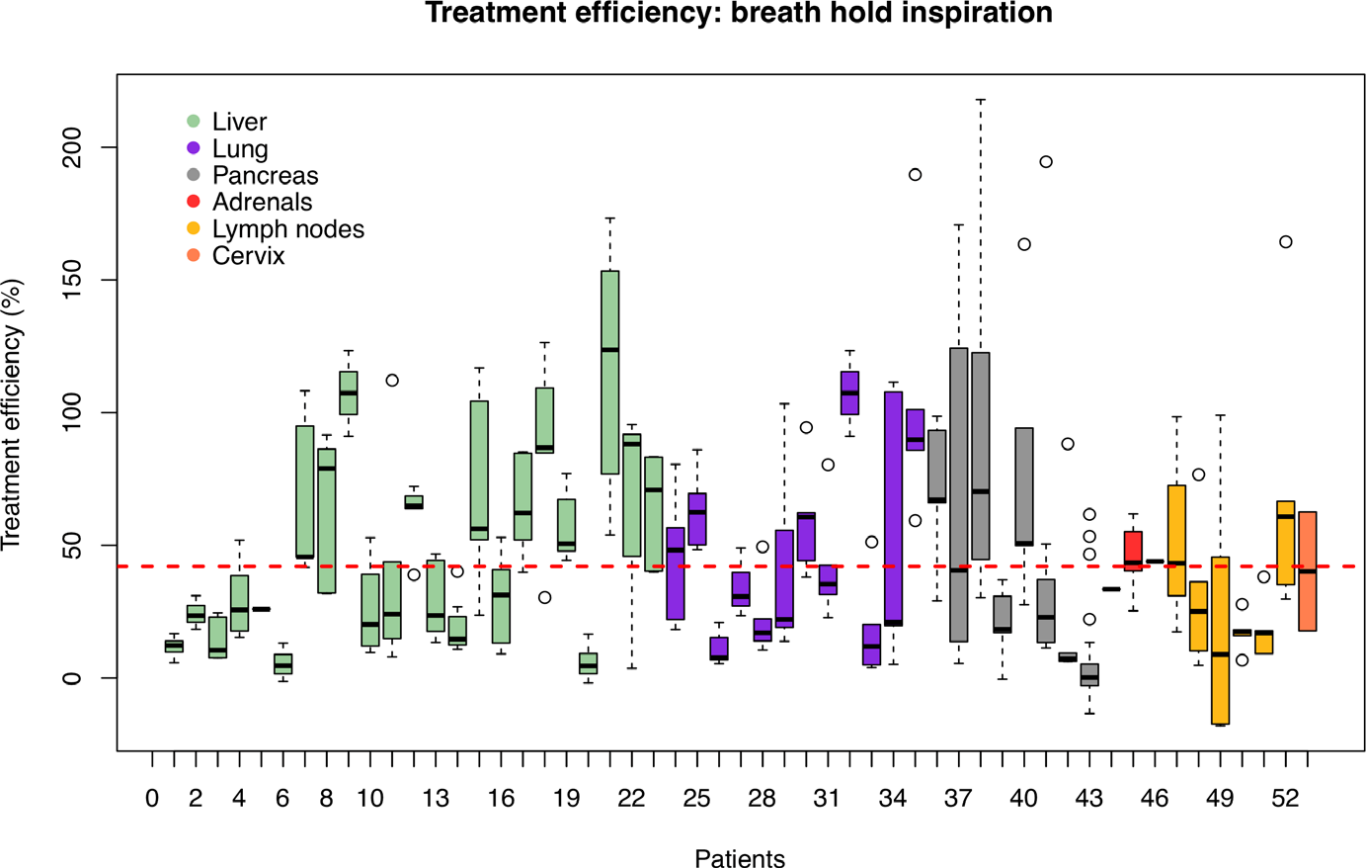
Patient‐specific treatment time efficiency for the group of patients treated with gating system (breath hold inspiration). Different treatment sites (liver, lung, pancreas, adrenal glands, lymph nodes, cervix) are reported in different colors.

**Fig. 3:**
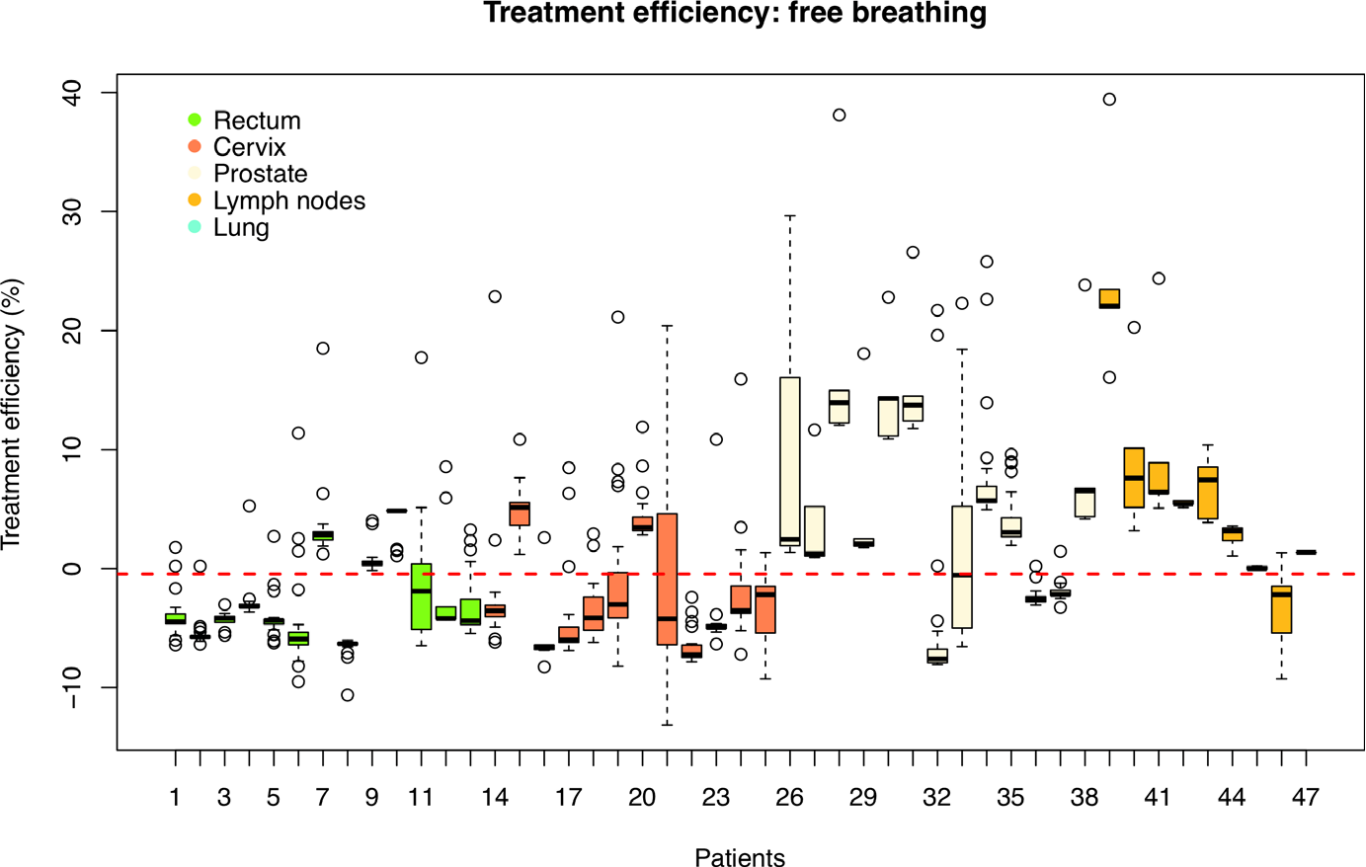
Patient‐specific treatment time efficiency for the group of patients treated in free breathing. Different treatment sites (rectum, cervix, prostate, lymph nodes, lung) are represented in different colors.

Treatment efficiency (*T_E_* (*X*,*V*,*R*)) results are also depicted in term of Whisker plot in Figs. 2 and 3 for patient’s group treated with (BHI) and without (FB) active breathing control approaches, respectively.

For BHI group, the highest mean *T_E_* (*X*,*V*,*R*) values is are for the lung patients (55.1%), the lowest for lymph node (32.6%) and the pancreas (35.1%) patients. Comparable *T_E_* (*X*,*V*,*R*) values have been found between liver (42.5%) and adrenal gland (43.4%) patients. It should be also considered the intra‐patient and intra‐site variability of the *T_E_* (*X*,*V*,*R*). For example, pancreas patients show the highest intra‐site *T_E_* (*X*,*V*,*R*) variability, also expressed by the highest value of the *T_E_* (*X*,*V*,*R*) standard deviation (50,2%), listed in Table 2. As well, pancreas patient (patient numbers 37 and 38) show the highest intra‐patient *T_E_* (*X*,*V*,*R*) variability. Lung and liver patients also show a relative variability of *T_E_* (*X*,*V*,*R*) both in terms of intra‐site (respectively 38.6% and 36.1% of *T_E_* (*X*,*V*,*R*) standard deviation) and of intra‐patients (patient number 21 and 22 for liver and 34 for lung).

On the other hand, for FB group the highest mean *T_E_* (*X*,*V*,*R*) values is for lymph node patients (7%), followed by prostate patients (2.8%). The other patients’ groups (excluding the lung, only one patient was included) show a negative mean *T_E_* (*X*,*V*,*R*) equal to −2.3% and −2.0% for the rectum and cervix patients respectively. Intra‐site variability is higher in lymph node and prostate (9.3% and 9.0% of *T_E_* (*X*,*V*,*R*) standard deviation respectively). Highest intra‐patient *T_E_* (*X*,*V*,*R*) variability, excluding some outliers, is visible in patient number 21 (cervix), 26 and 33 (prostate). Similar considerations and results can be translated to the other parameters included in the study (*T_Abo_* (*X*,*V*,*R*), *T_Attp_* (*X*,*V*,*R*), *T_TPSttp_* (*X*,*V*,*R*)) used to compute the *T_E_* (*X*,*V*,*R*).

**Fig. 4:**
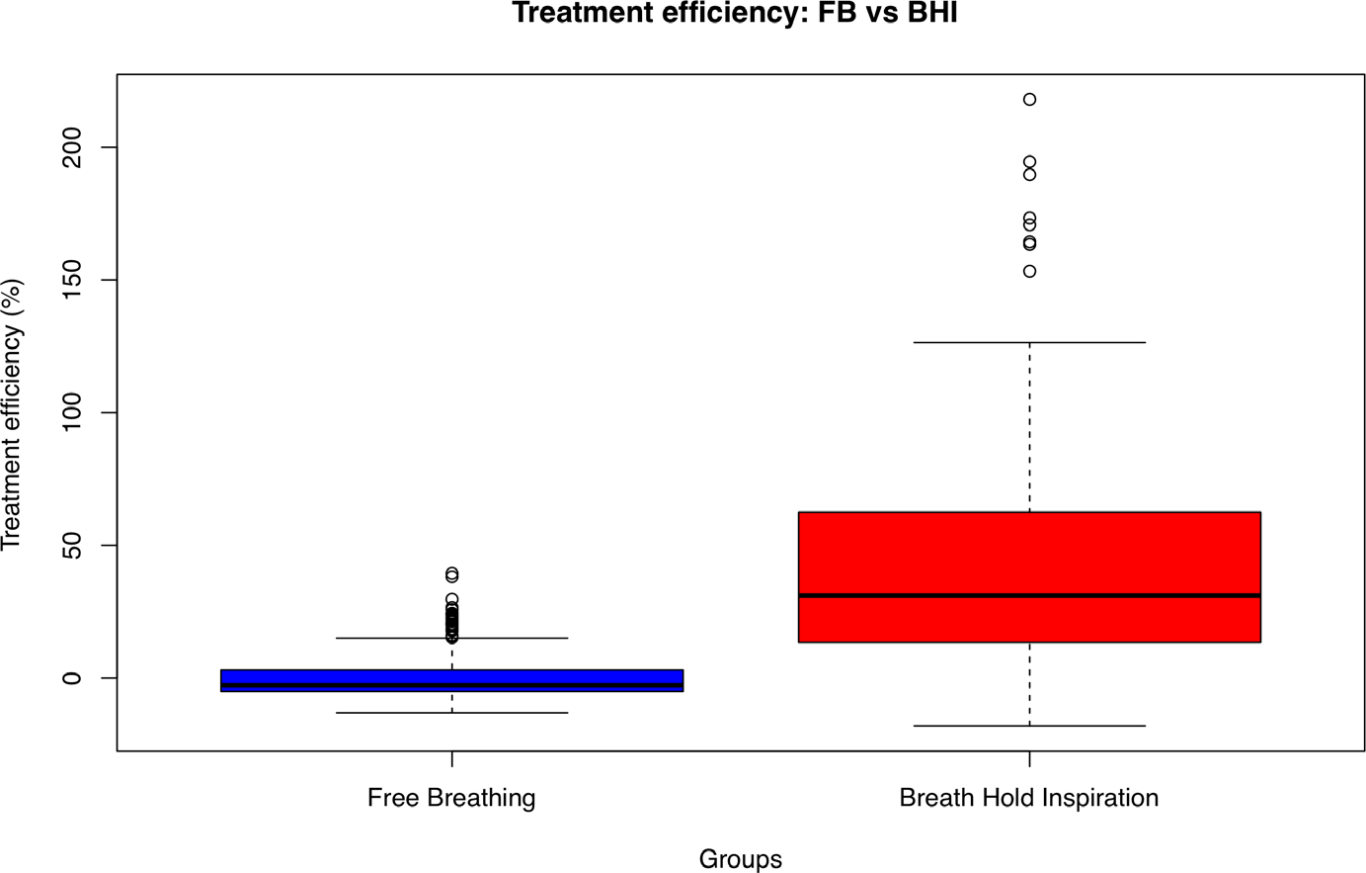
Overall treatment time efficiency for the group of patients treated in breath hold inspiration or free breathing conditions.

Overall *T_E_* (*X*,*V*,*R*) results of the two evaluated groups of patients are further summarized in Fig. 4, showing a statistically significant difference (*P* < 0.001) with a mean value of 42.4% and −0–5% for the BHI and FB group, respectively.

### 3.B Treatment slot time

The time required for the following PTP was evaluated (mean (min‐max)) in order to investigate the appropriateness of the foreseen treatment slot time occupancy:

- Patient ready for positioning: 5 min (3.5–6.5 min)
- Patient positioning: 4 min (3–5 min)
- Positioning correction: 4 min (1.5–7.5 min)
- Adaptive process: 30 min (15–60 min)

The average pretreatment time is 13 min for nonadaptive patients (14 and 12 for SBRT and long course treatment respectively), 43 min for adaptive ones. Table 2 describes actual total treatment process times (*T_Attp_* (*X*,*V*,*R*), while the actual treatment slot time occupancy (*T_Attp_* (*X*,*V*,*R*) + PTP) is described in Table 3.

**Table 3:** Actual treatment slot time occupancy is expressed, for patients treated with and without active breathing monitoring approach, in terms of *T_Attp_* (*X*,*V*,*R*) sum and the PTP is compared with the current daily treatment room schedule time slot for adaptive (ADP), SBRT and standard fractionation (long course) treatments.

Treatment room slot time	ADP (60 min)		SBRT (45 min)		Long course (30 min)	
	*T_Attp_* (*X*,*V*,*R*)	PTP	*T_Attp_* (*X*,*V*,*R*)	PTP	*T_Attp_* (*X*,*V*,*R*)	PTP
BHI	16.8 (8.2–36.3)	43.0	15.2 (7.6–38.2)	14.0	13.1 (6.9–22.5)	12.0
FB	9.0 (4.6–11.9)	43.0	13.7 (8.8–23.9)	14.0	11.7 (7.0–22.4)	12.0

Regarding the adaptive treatments, in 33 out of 96 (34.0%) of the delivered fractions, the scheduled time (*T_Attp_* (*X*,*V*,*R*) + PTP) was found to be not sufficient (>60 min). For SBRT treatment, only in six out 391 (1.5%) of the delivered fractions the scheduled time was found not sufficient (>45 min). Finally, for the long course treatment, only in 23 out of 528 (4.3%) of the delivered fraction the scheduled time was found not sufficient (<30 min).

### 3.C Clinical, planning and delivering parameters

Table 4 lists some relevant clinical, planning and delivery parameters for each category. The parameters that highlight a relevant difference between the two groups (in terms of mean value) are: number of fractions [44 (BHI) vs 130 (FB)]; dose per fraction [8,9 (BHI) vs 5,3 (FB)]; number of SBRT treatments [96% (BHI) vs 51% (FB)]; number of adaptive treatments [22% (BHI) vs 5% (FB)]; number of beams [9.9 (BHI) vs 12.0 (FB)]; number of segments [64.7 (BHI) vs 76.1 (FB)] and CTV volume [29.6 cc (BHI) vs 86.3 cc (FB)].

**Table 4:** Patients clinical, planning and delivering parameters for the patients of the considered groups.

Categories	Number of plans	Number of fractions	Dose per fraction (Gy)	ROI (%)	Boundary (mm)
			Mean (range)	Mean (range)	Mean (range)
BHI					
Lung	12	61	10.5 (7–14)	6.8 (4–18)	3.9 (3–5)
Liver	23	105	9.8 (5–12.5)	6.4 (2–13)	4.3 (3–5)
Pancreas	8	68	6.8 (1.8–10)	6 (5–10)	4 (3–5)
Adrenal gland	3	7	10	5.9 (4–8)	4.7 (3–5)
Lymph node	6	25	9.7 (4–12.5)	6.4 (5–10)	3.7 (3–5)
Cervix	1	2	5	7.5 (5–10)	3
FB					
Rectum	13	310	2.2	7.7 (5–12)	5
Cervix	12	117	2.1 (1.8–2.3)	8 (5–17)	5 (3–6)
Prostate	13	183	4.7 (2.7–7)	7.5 (5–20)	4.7 (3–5)
Lymph nodes	8	40	7.4 (4–10)	7.3 (5–20)	3.8 (3–5)
Lung	1	1	10	5	3

Categories	SBRT	Adaptive treatment	Number of beams	Number of segments	Number of completion fractions	CTV (cc)
			Mean (Range)	Mean (range)	Mean (range)	Mean (range)
BHI						
Lung	100%	0%	8.1 (6–10)	61.6 (27–96)	24	3.9 (0.2–13.4)
Liver	100%	0%	8.6 (6–12)	59.5 (32–80)	46	22.4 (0.9–241.2)
Pancreas	75%	62.5%	11.4 (8–18)	76.6 (55–100)	22	112.3 (4.1–281.3)
Adrenal gland	100%	33%	14.3 (11–18)	83 (50–100)	2	29.8 (20.8–33)
Lymph node	100%	37.5%	9.2 (8–12)	57.8 (40–70)	10	7.3 (0.2–16.3)
Cervix	100%	0%	8	50	1	1.9
FB						
Rectum	0%	0%	14.6 (8–15)	74.6 (60–100)	57	226.3 (32.4–925.8)
Cervix	0%	0%	11.4 (8–16)	86.4 (69–129)	71	101.2 (14.2–584.3)
Prostate	54%	0%	16.5 (8–16)	85.6 (58–128)	28	37.2 (1.5–51.2)
Lymph nodes	100%	25%	10.3 (7–16)	74.9 (51–130)	11	20.1 (0.5–49.5)
Lung	*100%*	*0%*	7	59	0	46.9

Possible correlations between *T_E_* (*X*,*V*,*R*) and the above mentioned parameters have been investigated. No statistically significant correlation has been found for the considered parameters and for both BHI and FB patients’ groups respectively.

## 4 DISCUSSION

MRgRT systems allow motion management during the radiotherapy, managing online target volume movements by means of an integrated gating system. Beam delivery is therefore allowed only if gating conditions satisfy the user defined pre‐set parameters. This could lead to a prolonged treatment time if the conditions to reproduce the gating condition^14^ are not quickly verified, essentially due to the patient’s anatomy variation^15^, ^16^ and compliance.^17^ Treatment room throughput and beam delivery efficiency are evidently related to these parameters and technical performance and represent important variables for daily treatment room schedule.

As already mentioned, our current daily treatment room schedule expects to reserve a 1‐h “patient in‐patient out” time slot for adaptive treatments, a 45‐min time slot for SBRT treatments and a 30‐min time slot for the remaining treatments. Nevertheless, to date, we do not differentiate the treatment slot time based on the gating or nongating beam delivery technique.

In this study we systematized treatment process time efficiency based on our clinical experience and suggest a new kind of approach to be implemented into the real‐time gating system already in use. This approach can be evaluated and verified in the MRgRT system since it enables real‐time tumor tracking and beam gating for both BHI and FB patients. While the FB treatment sites are generally different from the BHI treatment sites, this ability of the MRgRT system to beam‐gate both cohorts allows comparisons of the treatment process time efficiency with minimal bias. Even if with less evident advantages, also FB patients could benefit the real‐time tumor tracking and beam gating treatment delivery. For example, tumor tracking can prevent bulk motion of the patient on the treatment couch or detect any possible changes in the target position due to a different bladder filling.

The use of *T_E_* (*X*,*V*,*R*) can be a valid indicator to be used in daily clinical practice for gating MRI‐Linac activity optimization. Not surprisingly, as also shown in Fig. 4, a statistically significant difference (*P* < 0.001) was found between the *T_E_* (*X*,*V*,*R*) of BHI and FB groups, with a mean value of 42.4% and −0.5% respectively. As reported in the results section, *T_E_* (*X*,*V*,*R*) also highlighted relevant intra‐patient and intra‐site differences among the BHI and FB groups.

Based on the results listed in Table 2 and depicted in Figs. 2 and 3, it could be also possible to predict an additional time required to complete the actual total treatment process time (*T_Attp_* (*X*,*V*,*R*)) in comparison with the estimated time by the TPS (*T_TPSttp_* (*X*,*V*,*R*)). This time (*T_Attp_* (*X*,*V*,*R*)−*T_TPSttp_* (*X*,*V*,*R*)) is in average equal to 4.4 min for BHI group. Among the BHI group, the lung site shows the maximum required additional time (6.4 min). On the other hand, for FB group, no additional time is needed.

The results reported in Table 3, further highlight how the BHI group can potentially exceed the scheduled treatment room slot time, especially for adaptive treatment, suggesting to better evaluate the possibility to increase the treatment room slot time to avoid delays in the scheduling of the daily treatment. The choice of managing the treatment in BH leads different clinical advantages for the patient: first of all, the quality of the MR image acquired is superior in BH, as it is not influenced by motion artifacts. The superior image quality leads to a more accurate delineation of the target and organs at risk, allowing a more precise dose delivery, also reducing the GTV to PTV margins.

Clinical, planning and delivery parameters were also evaluated, as listed in Table 4, in order to better understand if *T_E_* (*X*,*V*,*R*) could be further optimized with these parameters, on the basis of the observed results. No significant dependence has been observed between time and these parameters.

As far as the authors know, this is the first study where treatment process time (*T_E_* (*X*,*V*,*R*)) has been quantitatively analyzed using data obtained from patients planning and treatment logs in order to evaluate treatment time when a gating system is used in the frame of a MRgRT treatment delivery. A similar experience was performed by Liu et al^17^ who developed an in‐house software tool to predict treatment delivery time for MRgRT (with ^60^Co MRIdian system). The proposed software tool was able to predict treatment delivery time with an average prediction error of 1.82% (0.22 min) and a maximal prediction error of 7.88% (0.89 min).

Liu and colleagues claimed that the accuracy of the proposed prediction algorithm was sufficient to support patient treatment appointment scheduling and was a reliable indicator for treatment plan complexity.^18^ Nevertheless, delivery time efficiency due to the gating system activation was not included in the proposed analysis.

As shown in our experience, *T_E_* (*X*,*V*,*R*) is a useful parameter able to successfully support the optimization of the treatment room scheduling, integrating gating related variables.

It has to be mentioned that patient compliance is an extremely variable parameter, difficult to quantify and also subject to important inter‐fraction *T_E_* (*X*,*V*,*R*) variability. In order to evaluate and quantify patient’s compliance, several protocols have been included in our clinical practice. Firstly, during the treatment preliminary medical examination, the radiation oncologist verifies the patient’s compliance by means of a dedicated questionnaire. In addition to general and clinical information, particular attention is given to MRI safe information, patient compliance and ability to breathing managing during treatment.^19^ Once the patient is eligible for MRgRT, a training session to verify and optimize the patient compliance and his/her ability in breathing management is performed during simulation.

As shown in Fig. S1, an evident and generalized learning curve is not feasible. BHI liver patients are probably the most stable group showing a minor inter‐fraction variation of the *T_E_* (*X*,*V*,*R*). Further analysis, probably with higher statistics, could definitely better investigate this issue.

One potential method to limit the intra‐fraction variability and reproducibility in breath hold treatments can be represented by the use of abdominal compression that, on the other side, maybe not compatible with MR‐Linac bore dimensions and coils placement.

A further option is the use of a high frequency percussive ventilation systems, able to induce apnea‐like suppression of respiratory motion and allow long enough breath hold duration to deliver long and complex RT treatment fractions, as reported by Peguret et al.^20^, ^21^ This kind of system could definitely support a reduction or at least a relevant optimization of the *T_E_* (*X*,*V*,*R*). Additionally, the use of this system could strongly support a relevant dose escalation if the stability of the target gating is reached. Further evaluation should be performed in MRgRT system environment.

The number of patients enrolled in this retrospective study is limited to the first 100 patients treated with the MRI‐Linac system, for a total of 919 fractions.

It should be also noticed that the *T_E_* (*X*,*V*,*R*) is strongly dependent on planning parameters: a learning curve on how to optimize them is foreseen, especially when a new TPS is implemented in clinical practice, as occurred in our Department with the transition from the MRIdian ^60^Co version to the MRI‐Linac one. In particular, in order not to compromise the dose distribution, *T_E_* (*X*,*V*,*R*) could have been potentially prolonged, as a reasonable trade‐off. Especially, the number of beams and the number of segments could significantly decrease the *T_Attp_* (*X*,*V*,*R*); on the other hand, ROI% could allow a better patient and site specific optimization of the gating system, successfully reducing the *T_E_* (*X*,*V*,*R*).

Even if the most common clinical indications are represented in the enrolled population, a selection bias can be recognized in our study, as patients affected by comorbidities that could affect their breathing cycle length and performance have been discarded due to the higher general fitness level required for MRI compatibility. Furthermore, diseases not primarily addressable to MRI‐Linac treatments have been directly excluded (i.e. H&N and brain), even if the use of gating systems in these anatomical sites is negligible.

Another limitation is the difference in treatment sites between the FB and BHI groups: nevertheless, it would have not been appropriate to use a BH technique for some of the FB sites or conversely. This is also because this retrospective analysis has been performed on each of the clinical delivered fractions for both FB and BHI patients and therefore is not feasible to compare FB and BHI approaches for the same treatment site, even prospectively since it would lead a suboptimal treatment for the patients.

Another limitation of this study is that patient repositioning time, in case of bulk motion or treatment position variation during delivery, has not been recorded. This circumstance may represent an unexpected source of treatment time extension that could impact on the time slot distribution throughout daily machine activity.

Finally, further technical and technological developments, such as novel tracking algorithms and higher frame per second real‐time cine MR imaging, could potentially affect *T_E_* (*X*,*V*,*R*) and this study can represent a robust baseline to compare future development.

## 5 CONCLUSIONS

This study quantitively evaluates the effect of gating efficiency in the MRgRT. The use of breath hold inspiration (BHI) gating technique can increase the treatment process time significantly, and treatment room time occupancy accordingly, up to a *T_E_* (*X*,*V*,*R*) = 218% (worst case scenario). More quantitative gating techniques should be considered to improve the use of MRgRT in terms of gating efficiency.

## CONFLICT OF INTEREST

The authors declare no conflict of interest.

## Supporting information


**Fig. S1**. Treatment efficiency for different treatment sites. (A) Inter‐fraction variability of treatment time efficiency for the group of patients treated with gating system (breath hold inspiration). Only four BHI sites have been considered (liver, pancreas, lymph node and lung) and only for patients with more than two treatment fractions.

## References

[1] World Health Organization . (2020). WHO report on cancer: setting priorities, investing wisely and providing care for all. World Health Organization. https://apps.who.int/iris/handle/10665/330745. License: CC BY‐NC‐SA 3.0 IGO.

[2] Borras JM , Lievens Y , Dunscombe P , et al. The optimal utilization proportion of external beam radiotherapy in European countries: an ESTRO‐HERO analysis. Radiother Oncol. 2015;116:38–44. 25981052 10.1016/j.radonc.2015.04.018

[3] Mutic S , Dempsey JF . The ViewRay system: magnetic resonance guided and controlled radiotherapy. Semin Radiat Oncol. 2014;24:196–199. 24931092 10.1016/j.semradonc.2014.02.008

[4] Van der Heide UA . MR‐guided radiation therapy. Phys Med. 2016;9:175.

[5] Cusumano D , Dhont J , Boldrini L , et al. Predicting tumour motion during the whole radiotherapy treatment: a systematic approach for thoracic and abdominal lesions based on real time MR. Radiother Oncol. 2018;129:456–462. 30144955 10.1016/j.radonc.2018.07.025

[6] Brandner E , Chetty I , Giaddui TG , Xiao Y , Huq MS . Motion management strategies and technical issues associated with stereotactic body radiotherapy of thoracic and upper abdominal tumors: a review from NRG oncology. Med Phys. 2017;44:2595–2612. 28317123 10.1002/mp.12227 PMC5473359

[7] Dieterich S , Green O , Booth J . SBRT targets that move with respiration. Phys Med. 2018;56:19–24. 30527085 10.1016/j.ejmp.2018.10.021

[8] van Sörnsen de Koste JR , Palacios MA , Bruynzeel AME , Slotman BJ , Senan S , Lagerwaard FJ . MR‐guided gated stereotactic radiation therapy delivery for lung, adrenal, and pancreatic tumors: a geometric analysis. Int J Radiat Oncol Biol Phys. 2018;102:858–866. 30061007 10.1016/j.ijrobp.2018.05.048

[9] Suzuki K , Gillin MT , Sahoo N , Zhu XR , Lee AK , Lippy D . Quantitative analysis of beam delivery parameters and treatment process time for proton beam therapy. Med Phys. 2011;38:4329–4337. 21859034 10.1118/1.3604153

[10] Suzuki K , Palmer MB , Sahoo N , et al. Quantitativeanalysis of treatment process time and throughput capacity for spot scanning proton therapy. Med Phys. 2016;43:3975–3976. 27370116 10.1118/1.4952731

[11] Yoshimura T , Shimizu S , Hashimoto T , et al. Big data analysis of treatment process time for the real‐time‐image gated‐spot‐scanning proton‐beam therapy (RGPT) system. Int J Radiat Oncol Biol Phys. 2018;102:e501–e502. 10.1002/acm2.12804 PMC7020995 31886616

[12] Placidi L , Romano A , Chiloiro G , et al. On‐line adaptive MR guided radiotherapy for locally advanced pancreatic cancer: clinical and dosimetric consideration. Tech Innov Patient Support Radiat Oncol. 2020;15:15–21. 32642565 10.1016/j.tipsro.2020.06.001 PMC7334416

[13] Tukey JW . Exploratory Data Analysis. Boston, MA: Addison‐Wesley; 1977.

[14] Cusumano D , Dhont J , Boldrini L , et al. Reliability of ITV approach to varying treatment fraction time: a retrospective analysis based on 2D cine MR images. Radiat Oncol. 2020;15:152 10.1186/s13014-020-01530-6. 32532334 PMC7291491

[15] Sonke JJ , Aznar M , Rasch C . Adaptive radiotherapy for anatomical changes. Semin Radiat Oncol. 2019;29:245–257. 31027642 10.1016/j.semradonc.2019.02.007

[16] Visser J , de Boer P , Crama KF , et al. Dosimetric comparison of library of plans and online MRI‐guided radiotherapy of cervical cancer in the presence of intrafraction anatomical changes. Radiat Oncol. 2019;14:126. 31300000 10.1186/s13014-019-1322-0 PMC6624982

[17] Klüter S , Katayama S , Spindeldreier CK , et al. First prospective clinical evaluation of feasibility and patient acceptance of magnetic resonance‐guided radiotherapy in Germany. Strahlenther Onkol. 2020;196:691–698. 32002567 10.1007/s00066-020-01578-z PMC7385000

[18] Liu S , Wu Y , Wooten HO , et al. Methods to model and predict the ViewRay treatment deliveries to aid patient scheduling and treatment planning. J Appl Clin Med Phys. 2016;17:50–62. 27074472 10.1120/jacmp.v17i2.5907 PMC5874812

[19] Boldrini L , Colloca GF , Villani E , et al. Magnetic resonance‐guided radiotherapy feasibility in elderly cancer patient: proposal of the MASTER scoring system. Tumori. 2020;15:300891620920709. 10.1177/0300891620920709 32410505

[20] Péguret N , Ozsahin M , Zeverino M , et al. Apnea‐like suppression of respiratory motion: first evaluation in radiotherapy. Radiother Oncol. 2016;118:220–226. 26979264 10.1016/j.radonc.2015.10.011

[21] Audag N , Van Ooteghem G , Liistro G , Salini A , Geets X , Reychler G . Intrapulmonary percussive ventilation leading to 20‐minutes breath‐hold potentially useful for radiation treatments. Radiother Oncol. 2019;141:292–295. 31668897 10.1016/j.radonc.2019.09.024

