# Supplementary material for: Quantitative analysis of MRI‐guided radiotherapy treatment process time for tumor real‐time gating efficiency
Source: J Appl Clin Med Phys. 2020 Oct 22;21(11):70–9. doi: 10.1002/acm2.13030 (PMC7701108; doi:10.1002/acm2.13030)
Supplement: Supplementary file 1 — Fig. S1. Treatment efficiency for different treatment sites. (A) Inter‐fraction variability of treatment time efficiency for the group of patients treated with gating system (breath hold inspiration). Only four BHI sites have been considered (liver, pancreas, lymph node and lung) and only for patients with more than two treatment fractions. [file ACM2-21-70-s001.docx]

**Supplementary Materials**


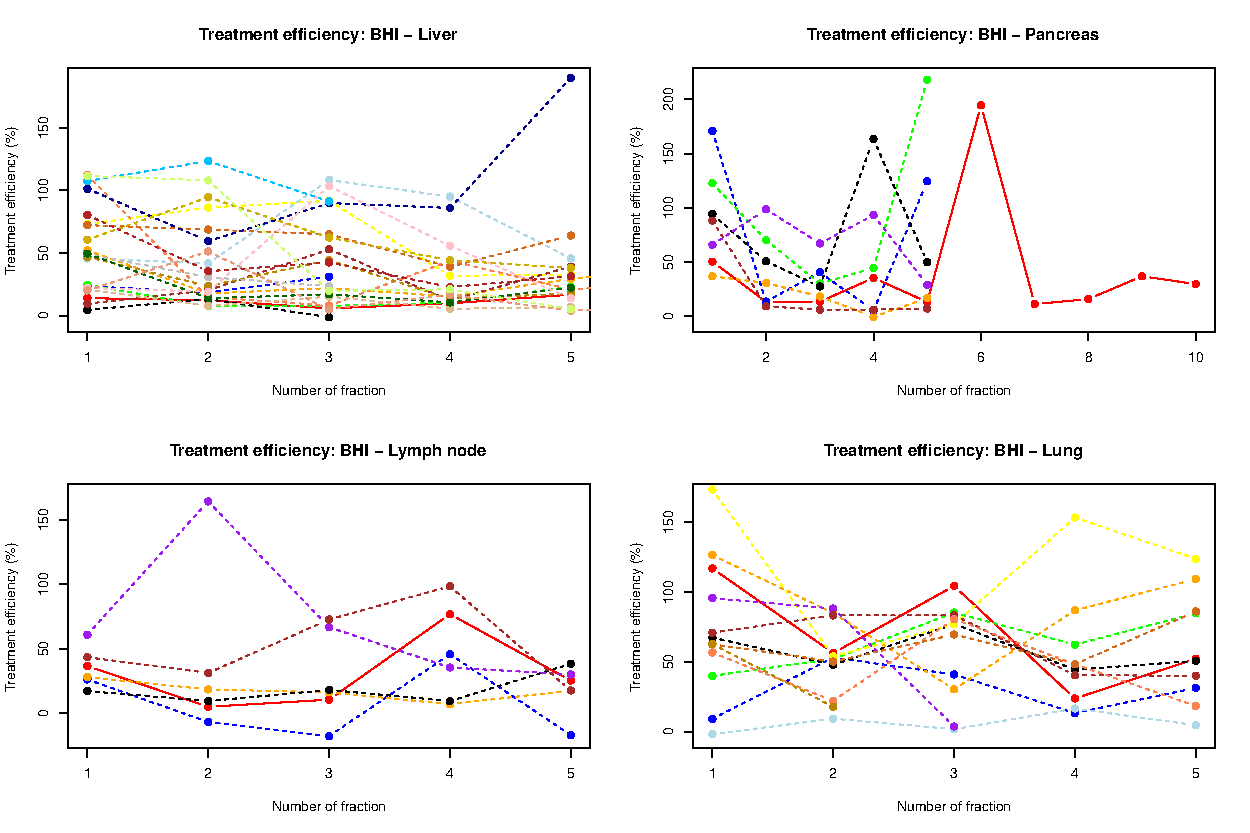


Figure 1 – Treatment efficiency for different treatment sites. (A) Inter-fraction variability of treatment time efficiency for the group of patients treated with gating system (breath hold inspiration). Only four BHI sites have been considered (liver, pancreas, lymph node and lung) and only for patients with more than two treatment fractions.
